# Oral rehydration solution for the management of fluid and electrolyte disturbances in patients with an ileostomy: A scoping review

**DOI:** 10.1002/jpen.70050

**Published:** 2026-01-09

**Authors:** Austin J. Hoeg, Jessica Jaques, Remy Johnson, Megan Kocher, Alexa Weingarden, Byron Vaughn, Alexander Khoruts, Cyrus Jahansouz, Ryan T. Hurt, Levi M. Teigen

**Affiliations:** ^1^ School of Graduate Medical Education, Mayo Clinic Rochester Minnesota USA; ^2^ Medical School University of Minnesota Minneapolis Minnesota USA; ^3^ University of Minnesota Libraries St. Paul Minnesota USA; ^4^ Department of Medicine, Division of Gastroenterology, Hepatology, and Nutrition University of Minnesota Minneapolis Minnesota USA; ^5^ Department of Surgery, Division of Colon and Rectal Surgery University of Minnesota Minneapolis Minnesota USA; ^6^ Division of General Internal Medicine, Mayo Clinic Rochester Minnesota USA; ^7^ Department of Food Science and Nutrition University of Minnesota St. Paul Minnesota USA

**Keywords:** enteral formulas, enteral nutrition, fluids, electrolytes/acid‐base, GI access, internal medicine, nutrition, nutrition support teams, nutrition support practice, outcomes research/quality, gastroenterology, renal disease, research and diseases, surgery

## Abstract

Ileostomy creation is common among patients with colorectal cancer, diverticulitis, and inflammatory bowel disease. Surgical removal or diversion of the colon can result in nutrition complications, most notably dehydration—a leading cause of hospital readmission—and increased risk of kidney injury. Existing studies on fluid and electrolyte management after ileostomy largely focus on short bowel syndrome or high‐output stomas, overlooking the physiological implications of ileostomy creation, even in patients with normal output. Given the absence of standardized oral rehydration protocols and limited interventional data, a scoping review was conducted to map the current evidence on oral rehydration solutions for postoperative fluid and electrolyte management in adults without short bowel syndrome or high‐output stoma. Comprehensive searches across five databases identified 5738 articles, of which 6 met inclusion criteria. Eligible studies examined adults with ileostomy for any standard indication in which oral rehydration solution use was a study objective. The included studies were methodologically heterogeneous, encompassing randomized controlled, crossover, double‐blinded comparator, retrospective cohort, and qualitative designs. Collectively, available evidence supports a pivotal role for oral rehydration solutions in both short‐term and long‐term management after ileostomy, improving hydration and reducing complications. This review highlights the need for standardized, evidence‐based oral rehydration regimens and underscores opportunities to reduce postoperative morbidity, kidney injury, and hospital readmissions while optimizing long‐term outcomes in this growing patient population.

## INTRODUCTION

Ileostomy creation is a standard procedure among patients with colorectal cancer, diverticulitis, and inflammatory bowel disease. Although precise global epidemiologic data on ileostomy prevalence are limited because of variations in reporting and healthcare systems, it is estimated that 750,000 to 1 million individuals in the United States are living with an ostomy, with approximately 150,000 new ostomies created each year.^1^

Although the colon is not essential for life, it plays a crucial—although often underrecognized—role in maintaining fluid and electrolyte balance.^2^ Consequently, surgical procedures that divert or remove the colon (eg, colectomy, proctocolectomy) are associated with an increased risk of dehydration and electrolyte disturbances. In patients with ileostomy, the loss of colonic absorptive function correlates with higher rates of fluid and sodium depletion, particularly during the early postoperative period. This physiological vulnerability is reflected clinically, as 30‐day and 60‐day hospital readmission rates for dehydration after ileostomy have previously been shown to range from 2.1%–13.2% (mean, 5.0%) and 7.3%–14.1% (mean, 10.3%),^3^ respectively, illustrating a clear relationship between altered intestinal physiology and adverse fluid balance outcomes.

Creation of ileostomy remains an essential component of surgical management for a variety of gastrointestinal diseases and operative contexts. A diverting loop ileostomy is commonly fashioned to protect a distal colorectal or coloanal anastomosis, particularly in the setting of rectal cancer after neoadjuvant therapy in which anastomotic healing is at increased risk.^4^ End ileostomy may be created following total colectomy for ulcerative colitis, emergent colectomy for perforation or fulminant colitis, or resection of the right colon for severe pathology. Historically, the goal following colectomy in ulcerative colitis was often a restorative proctocolectomy with ileal pouch‐anal anastomosis, a staged procedure dependent on temporary diversion.^5^ However, the use of pouches has declined nationally, in part because of the effectiveness of modern biologic therapies in inflammatory bowel disease.^6^ Regardless of the indication or ultimate surgical end point, the expectation remains that the ileum will adapt to compensate for some of the absorptive and fluid regulatory functions of the colon, although this adaptation is inherently limited.^7^, ^8^

Intestinal adaptation is the natural compensatory process by which the body enhances fluid and nutrient absorption following resection. Data on human bowel adaptation following resection are sparse. Still, long‐term ileostomy output has been shown to correlate closely with body size and with a mean daily ileostomy output of approximately 600 ml.^9^, ^10^ The site and extent of resection, luminal stimulation with nutrients, and intestinotrophic growth factors all influence intestinal adaptation. Structural adaptations can include hyperplasia, angiogenesis, as well as bowel dilation and elongation. Mucosal changes may include an increase in cell transporters, accelerated crypt cell differentiation, reduced transit time, and enhanced absorption of nutrients and fluids.^11^ Most adaptation occurs within the first 2 years following surgery. However, recent data have shown that adaptation can happen beyond this time point.^12^

Fluid and electrolyte management are well understood to be critical in the presence of short bowel syndrome and high‐output stoma. In brief, short bowel syndrome can occur after a substantial resection of the small bowel. High‐output stoma is a significant postoperative complication following ileostomy. Generally defined as stomal output exceeding 1500–2000 ml per day for ≥2 consecutive days, high‐output stoma occurs in approximately 16%–50% of patients in the early postoperative period.^8^, ^13^ Crohn disease, infections, prokinetic agents (eg, metoclopramide), and the use of other medications (eg, proton pump inhibitors) can also exacerbate stoma output over time.^14^, ^15^, ^16^ Although the majority of clinically significant dehydration and electrolyte abnormalities occur in patients with high‐output stomas, one cross‐sectional study found that subclinical sodium depletion may be present in up to 45% of patients with an ileostomy without high output.^17^ As fluid and electrolyte management of short bowel syndrome and high‐output stoma has been reviewed elsewhere,^12^, ^18^ it will not be the focus of this review.

In patients undergoing ileostomy, the impact of targeted interventions, such as oral rehydration solution, on clinical end points, including dehydration, is incompletely understood. Therefore, this scoping review aims to capture evidence on the fluid and electrolyte management of ileostomies, to identify gaps in current knowledge, inform best practices, and direct future research to improve both short‐term and long‐term outcomes for this vulnerable patient population, including prevention of acute kidney injury and chronic kidney disease.

### Renal implications of ileostomy creation

#### Acute kidney injury

Under normal conditions, the colon reabsorbs a substantial amount of sodium and water; however, following ileostomy, these functions are lost, and although the ileum can compensate through intestinal adaptation,^19^ patients are left vulnerable to dehydration. In the immediate postoperative period, ileostomies can produce over 1.5–2 L of fluid per day, leading to significant sodium and water losses. Subsequent dehydration and a decrease in adequate circulating volume impair the kidney's ability to clear nitrogenous waste products (eg, urea, creatinine), leading to prerenal azotemia.^8^, ^14^ If left uncorrected, persistent hypoperfusion can lead to ischemic injury of the renal tubules, resulting in acute tubular necrosis—a form of intrinsic acute kidney injury.^20^ The tenuity of this physiologic cascade underscores the importance of closely monitoring and addressing changes in volume status during the early postoperative phase, in particular.

#### Chronic kidney disease

Although chronic kidney disease is often associated with systemic diseases like diabetes mellitus and hypertension, gastrointestinal surgical interventions such as ileostomy can also increase long‐term risk.^21^ Several cohort studies have identified a disproportionately high incidence of chronic kidney disease among patients with ileostomies, particularly those who experience recurrent dehydration and acute kidney injury episodes in the early postoperative period.^22^, ^23^ One cohort study found that the risk of progression to new or worse chronic kidney disease in patients with ileostomies was double that of those who had undergone rectal resection without an ileostomy (16.9% vs 8.2%).^23^ Repeated insults to the renal parenchyma from unresolved acute kidney injury episodes may contribute to nephron loss and fibrosis, driving progression to chronic kidney disease. Moreover, chronic low‐grade volume depletion can sustain a state of renal hypoperfusion, exacerbating kidney injury over time.^21^ Thus, the burden placed on the kidneys by altered fluid dynamics following ileostomy creation may underlie a long‐term predisposition to chronic kidney dysfunction.

### Potential of oral rehydration solution to support volume and electrolyte status after ileostomy creation

**Figure 1:**
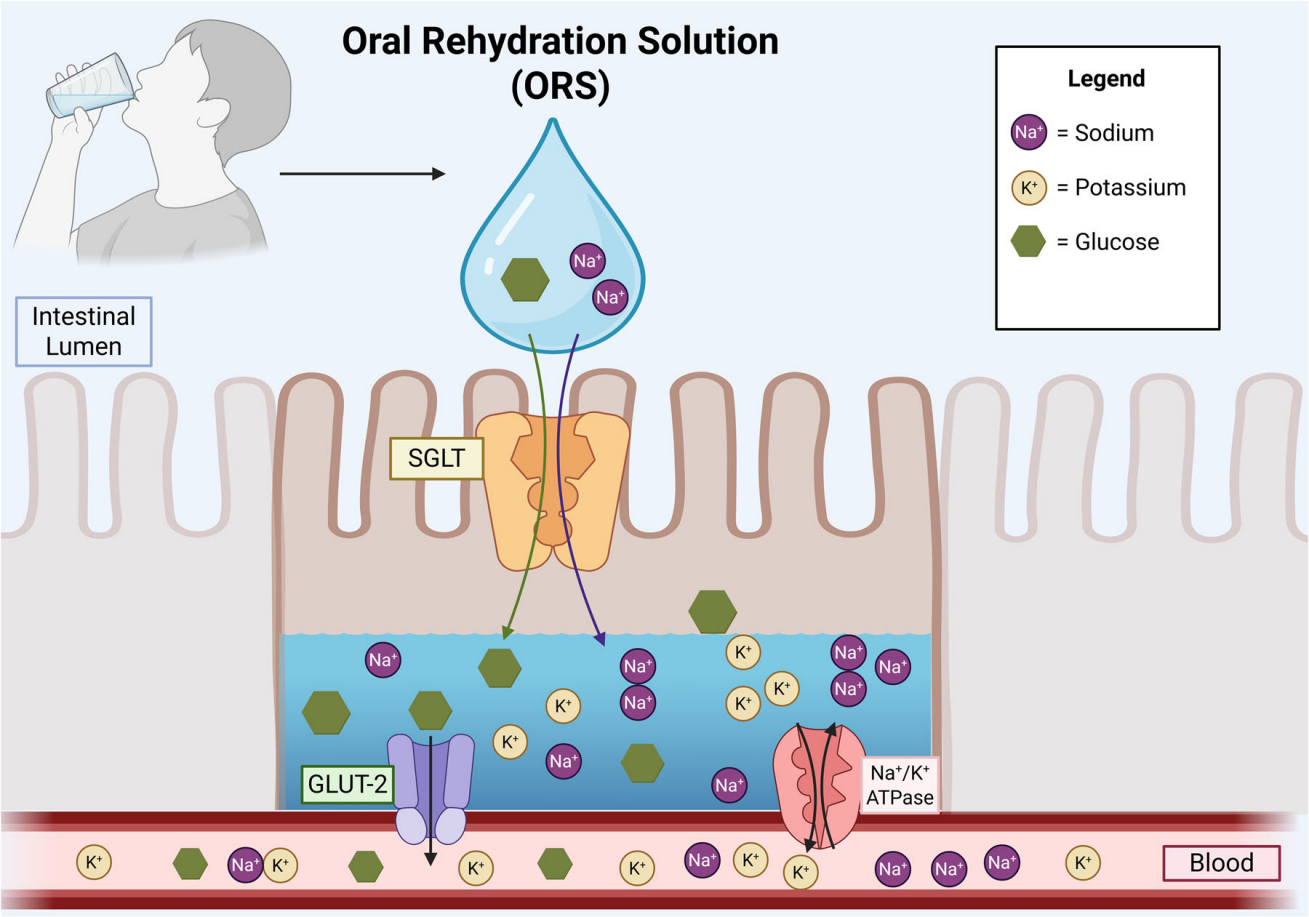
Use of the sodium‐glucose cotransporter with oral rehydration solutions to optimize fluid absorption. The rational underlying oral rehydration solutions (ORS) is that by taking advantage of the intestinal sodium‐glucose cotransporter (SGLT), uptake of glucose and sodium result in an osmotic gradient that facilitates water absorption.

We posit that early postoperative oral rehydration solution administration in patients undergoing ileostomy is a practical, feasible, and cost‐effective option for preventing dehydration, acute kidney injury, and chronic kidney disease. By leveraging the preserved sodium‐glucose cotransport in the small intestine (Figure 1),^24^ oral rehydration solution may serve as a more effective alternative to oral hypotonic solutions (eg, water) and a more cost‐effective alternative to intravenous isotonic solutions (eg, normal saline) to prevent dehydration and sodium depletion, ultimately mitigating the risk of prerenal azotemia and progression to chronic kidney disease. Thus, we postulate that early postoperative oral rehydration solution may be an underused yet cornerstone intervention for renal protection in this population.

## METHODS

The objectives, inclusion criteria, and methods for this scoping review were prespecified and published in a protocol with the Open Science Framework. We used a previously established scoping review methodology to guide our study methods and applied the Preferred Reporting Items for Systematic Reviews and Meta‐Analyses for Scoping Reviews (PRISMA‐ScR).

### Search strategy, literature sources, and supplementary data

A comprehensive search strategy was adapted from a previous protocol, run by a science librarian (MK) in collaboration with subject matter experts (LT and AH) and tested against a set of exemplar articles.^25^ Searches were run in five databases: Medline via Ovid, Agricola via Ovid, Embase Classic and Embase via Ovid, Scopus via Elsevier, and the Cochrane Library via Wiley. No date or language limits were applied in the databases. Limits were applied to remove Medline results from databases other than Medline. Searches were run on March 10, 2025. Full search strategies are available in Appendix A.

### Inclusion criteria

Inclusion criteria were defined based on the Patient, Concept, and Context criteria outlined by the Joanna Briggs Institute methodology for scoping reviews. These criteria encompassed any papers directly discussing adults who underwent ileostomy for any recognized treatment indication, with perioperative nutrition management as a study objective, including relevant perioperative alimentation. According to the Effective Practice and Organisation of Care reviews criteria, the following study designs were considered for inclusion: randomized controlled trials, nonrandomized controlled trials, controlled before‐and‐after studies, and interrupted time series. Case reports and case series were also considered for inclusion. Non‐English publications presented with an English language abstract were included if they had sufficient evidence for extraction. Notably, both quantitative and qualitative studies from each of these categories were included. Reviews (eg, meta‐analyses, systematic reviews, narrative reviews), chapters and books, gray literature (dissertations, reports, and conference proceedings), unpublished, non–peer‐reviewed articles, and all other nonoriginal articles were excluded.

**Figure 2:**
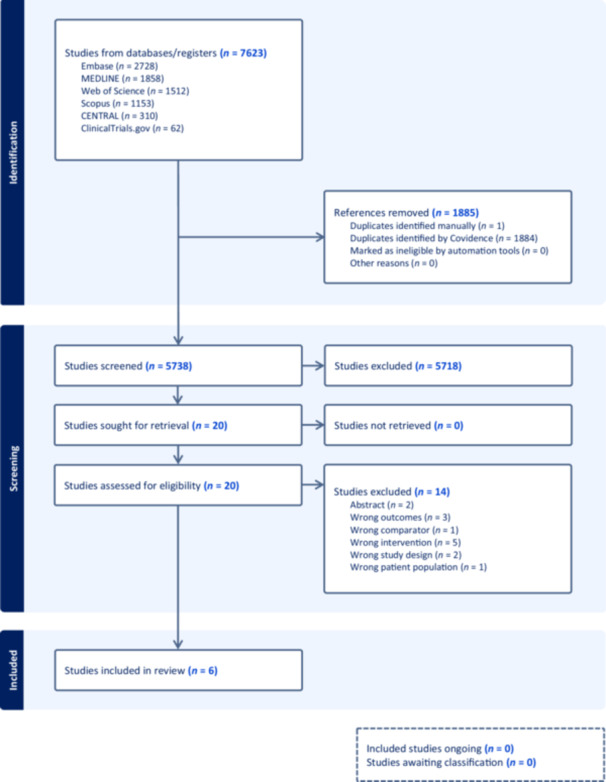
Preferred Reporting Items for Systematic Reviews and Meta‐Analyses (PRISMA) flow diagram outlining the article selection process.

Results from the literature search (Figure 2) were uploaded to Covidence, a web‐based collaboration software platform that streamlines the production of systematic and other types of literature reviews. Duplicate references were removed to the extent possible, and the articles were screened for eligibility based on the inclusion and exclusion criteria before data extraction.

The abstract stage inclusion criteria were any study that discussed some variation of the term “ileostomy” and “oral rehydration solution” (eg, oral rehydration salts, glucose‐salt solution). Article titles and abstracts were independently reviewed for eligibility by two coders (AH and JJ) out of a 10‐person research team. Titles and abstracts must have discussed ileostomy and clearly indicated their interest in perioperative nutrition management as a study objective while reporting on clinical outcomes in the findings section. Once references were determined to be potentially eligible, full articles were obtained for studies that appeared to meet the eligibility criteria based on title and abstract screening. Next, the full text was independently reviewed by two coders for eligibility and inclusion. First through third authors (AH, LT, and JJ) assumed this role and completed the title and abstract and full‐text screening. At both the abstract and full‐text stages, disagreements were resolved by bringing the senior author (LT) in for discussion if necessary.

### Data extraction, synthesis, and analysis

An a priori charting table was developed by two reviewers (AH and LT). The charting table consisted of predefined data items for extraction, which included title, authors, publication year, age, sex, race and/or ethnicity, surgical indication, type of surgery, perioperative nutrition management modality, any associated clinical outcomes, as well as demographic information regarding patient and study characteristics. The charting form was revised iteratively as each of the included articles was screened. Two reviewers (AH and JJ) extracted data from the original search; the first reviewer then independently verified the data (AH). Any discrepancies were resolved through a rereview of the study or a discussion with the senior author (LT).

### Patient and public involvement

No patients were involved.

## RESULTS

As outlined in the PRISMA flow diagram (Figure 2), 7623 records were identified through database searches. After removal of 1885 duplicates, 5738 studies underwent title and abstract screening. Of these, 20 met criteria for full‐text review based on predefined inclusion and exclusion parameters. Ultimately, five original studies^26^, ^27^, ^28^, ^29^, ^30^ and one secondary analysis^31^ were included, yielding six publications summarized in Table 1.

**Table 1:** Overview of all studies included.

Author and location	Design	*n*	Mean age, years	Procedure indication, *n* (%)	Time‐to‐intervention	Length	Intervention considerations	ORS composition	ORS volume	Primary outcomes	Secondary outcomes
Rud et al,^29^ Denmark	Randomized, double‐blinded, active comparator‐controlled 3 × 3 crossover study	13	52 (averaged median)	Ulcerative colitis: 4 (31) Crohn disease: 4 (31) Cancer: 1 (8) Diverticulitis: 1 (8) Hirschsprung disease: 1 (8) Sigmoid volvulus: 1 (8) Constipation: 1 (8)	Long‐term postileostomy; 16 years (averaged mean)	4 weeks (±1 week)	Not to ingest any solids or liquids for at least 30 min before and after ingesting ORS	(1) Whey protein isolate (Na, 34–45 mmol/L mEq/L; osmolality, 248–270 mOsm/kg; glucose, 32 g/L; protein, 40–48 g/L) (2) Caseinate protein (Na, 34–45 mmol/L mEq/L; osmolality, 248–270 mOsm/kg; glucose, 26 g/L; protein, 40–48 g/L) (3) Whey protein hydrolysate (Na, 34–45 mmol/L mEq/L; osmolality, 248–270 mOsm/kg; glucose, 22 g/L; protein, 40–48 g/L)	500 ml/day (250 ml twice daily)	Ileostomy output (wet weight)	(1) Fecal Na, K, Mg, Ca, energy content, and dry weight (2) Urine volume, Na, K, Mg, osmolality, creatinine, and carbamide (3) Plasma Na, K, Mg, osmolality, aldosterone, creatinine, carbamide, CTx, and P1PN (4) eGFR (5) Total venous carbon dioxide (6) Dietary intake (7) Palatability (8) Body weight and composition (9) HGS (10) 30‐s S2S count (11) Fatigue (12) Cognitive function
Kudoh et al,^26^ Japan	Prospective randomized crossover	Group A: 9 Group B: 11	Group A: 30 ± 12 Group B: 40 ± 12	Ulcerative colitis: 20 (100)	Immediate postileostomy; 2–3 weeks	2 weeks	(1) Ad libitum solids and liquids (2) No IV fluid supplementation (3) No antidiarrheal agents	Composition (Na, 50 mEq/L; K, 20 mEq/L; Cl, 50 mEq/L; Mg, 2 mEq/L; P, 2 mmol/L; lactate, 31 mEq/L; glucose, 1.8%)	1000 ml/day	(1) Urine (osmotic pressure, Na, K, Cl, and creatinine) (2) Plasma (renin, aldosterone, vasopressin, osmotic pressure, and creatinine)	Qualitative questionnaire (thirst, fatigue, appetite, sweat, dizziness, abdominal pain, and abdominal discomfort)
Rud et al,^28^ Denmark	Randomized, double‐blinded, active comparator‐controlled 2 × 2 crossover study	8	59	Ulcerative colitis: 5 (62.5) Crohn disease: 2 (25) Cancer: 1 (12.5)	Long‐term postileostomy; 11.5 years (averaged median)	2 days	(1) Not to ingest any solids or liquids for at least 60 min before and after ingesting ORS (2) Maintain habitual dietary intake except for substituting 800 ml of their daily fluid intake with the intervention supplement when directed (3) Reduce intake of milk or water by 800 ml/day when substituting habitual beverages	(1) Iso‐osmolar (energy, 142 kJ/100 g; water, 91%; osmolality, 276 mOsm/kg; sucrose, 1.45%, 42 mmol/L; sucralose [0.001%, 0.03 mmol/L]; hydrolyzed whey protein, 4.5%; cocoa powder, 3.42%) (2) Hyperosmolar (energy, 238 kJ/100 g; water, 84%; osmolality, 681 mOsm/kg; sucrose, 1.45%, 42 mmol/L; glucose, 6.25%, 347 mmol/L; hydrolyzed whey protein, 4.5%; cocoa powder, 3.42%)	800 ml/day (200 ml four times daily)	Ileostomy output (wet weight)	(1) Urine (volume, sodium, and osmolality) (2) Plasma (sodium, osmolality, and creatinine) (3) Estimated intakes of fluids (all beverages) (4) Total water intake (5) Energy intake (kJ/day) (6) Total food and fluid intake (g/day) (7) Sodium intake (mmol/day)
Westfall et al,^30^ USA	Retrospective cohort	312	59.97 ± 15.09	Ulcerative colitis: 34 (10.9) Crohn disease: 39 (12.5) Cancer: 74 (23.71) Diverticulitis: 135 (43.27) Other: 30 (9.62)	Immediate postileostomy; 0–1 days (postoperatively)	36 days	Review of inputs and outputs, antimotility and appliance needs, and trained nurse reeducation 4–7 days after discharge, 30 days postoperatively, and every 1–2 weeks thereafter as needed	Composition (NaCl, 2.6 g; KCl, 1.5 g; glucose anhydrous, 13.5 g; trisodium citrate dehydrate, 2.9 g)	946 ml/day	Number (%) of readmissions because of dehydration and/or AKI	(1) ED visits because of AKI and/or dehydration (2) ED visits and readmissions because of other common readmission diagnoses (eg, ileus, surgical site infection) (3) Postoperative complications (4) Time to readmission for each readmission diagnosis (5) Inpatient and postdischarge stoma output (6) Antimotility agent and home IV fluid needs (7) Stoma reversal rates (8) Hospital postoperative length of stay
Migdanis et al,^27^ Greece	Randomized controlled trial	IG: 39 CG: 41 NIG: 37	65 ± 12	Ulcerative colitis: 5 (4.27) Crohn disease: 4 (3.42) Cancer: 105 (89.75) Diverticulitis: 3 (2.56)	Immediate postileostomy; 0–1 days (postoperatively)	40 days	Restrict both hypotonic (eg, tea, coffee, water, and reduced sugar fruit juices and squash) and hypertonic (eg, fruit juices, cola, and sip feeds) fluids to 1 L/day	Composition (NaCl, 3 g; sodium citrate, 2 g; glucose, 22 g; magnesium citrate, 3.5 g; beta‐carotene, 1 g; orange aroma extract, 1 g)	1000 ml/day	(1) Serum (Na, K, Mg, P, serum albumin level, total protein, creatinine, and urea) (2) eGFR	(1) Hospital readmission (2) Mean ileostomy output (ml/day)
Bradley et al,^31^ USA	Qualitative	17	<20–70+ (age range)	Ulcerative colitis: 5 (4.27) Crohn disease: 4 (3.42) Cancer: 105 (89.75) Diverticulitis: 3 (2.56)	Immediate postileostomy; 0–1 days (postoperatively)	36 days	Review of inputs and outputs, antimotility and appliance needs, and trained nurse reeducation 4–7 days after discharge, 30 days postoperatively, and every 1–2 weeks thereafter as needed	Composition (NaCl, 2.6 g; KCl, 1.5 g; glucose anhydrous, 13.5 g; trisodium citrate dehydrate, 2.9 g)	946 ml/day	Barriers and facilitators to compliance with ORS kit instructions	None

Abbreviations: AKI, acute kidney injury; Ca, calcium; CG, control group; Cl, chloride; CTx, carboxy‐terminal collagen crosslinks; ED, emergency department; eGFR, estimated glomerular filtration rate; HGS, handgrip strength; IG, intervention group; IV, intravenous; KCl, potassium chloride; K, potassium; Mg, magnesium; Na, sodium; NaCl, sodium chloride; NIG, nonintervention group; ORS, oral rehydration solution; P, phosphorus; P1PN, procollagen I intact N‐terminal; S2S, sit‐to‐stand.

The included studies examined ileostomy created for inflammatory bowel disease (ulcerative colitis^26^, ^27^, ^28^, ^29^, ^30^, ^31^ and Crohn disease^27^, ^28^, ^29^, ^30^, ^31^), cancer, Hirschsprung disease,^32^ diverticulitis,^27^, ^29^, ^30^, ^31^ sigmoid volvulus,^29^ and constipation.^28^ One study listed an “Other” category without specifying conditions.^30^ Study designs were heterogeneous, including both observational and interventional trials with varying degrees of randomization and blinding. The composition, dosing, and administration of oral rehydration solution, as well as adjunctive dietary parameters and outcome measures, differed across studies. Collectively, these studies explored nutrition management immediately following ileostomy creation.

### Oral rehydration solution improves biochemical indicators of hydration status and kidney function

Four studies reported biochemical indicators of hydration^26^, ^27^, ^28^, ^29^ (Table 2).

**Table 2:** Biochemical indicators.

Biochemical indicator	Author					
	Kudoh et al^26^, a	Kudoh et al^26^, a	Migdanis et al^27^	Rud et al^28^, b	Rud et al^b^	Rud et al^29^, ^b^
	Intervention					
	**Group A (ORS days 1–7, MW days 8–14)**	**Group B (MW days 1–7, ORS days 8–14)**	**Post–non‐ORS vs post‐ORS**	**Δ (baseline vs post‐ORS)**	**Iso‐osmolar vs hyperosmolar ORS**	**Δ (baseline vs post‐ORS)**
Urine volume, mean (*P* value), ml/day	Day 0: 569 (data not available) Day 7: 616 (data not available) Day 14: 589 (data not available)	Day 0: 911 (data not available) Day 7: 1449 (data not available) Day 14: 1283 (data not available)	Data not collected	Iso‐osmolar: +305 (0.02) Hyperosmolar: −35 (0.81)	Δ: +470 (0.02)	Whey isolate: −194 (0.07) Caseinate: +46 (0.62) Whey hydrolysate: +33 (0.76)
Urine sodium^a,b^, mean (*P* value)	Day 0: 15.2 (data not available) Day 7: 59.4 (0.005 [Δ day 0–7]) Day 14: 32.6 (data not available)	Day 0: 17.4 (0.39 [Δ day 0–7]) Day 7: 59.4 (0.012 [Δ day 7–14]) Day 14: 103 (0.001 [Δ day 0–14])	Data not collected	Iso‐Osmolar: +20 (0.08) Hyperosmolar: −7 (0.09)	Δ: +36 (0.02)	Whey isolate: 1.25 (0.68) Caseinate: 1.20 (0.68) Whey hydrolysate: 1.09 (0.79)

Abbreviations: Δ, change from baseline to post‐ORS; MW, mineral water; ORS, oral rehydration solution; SSI, surgical site infection.

^a^ Measured urine sodium (mmol/day).

^b^ Measured urine sodium (mEq/g CRE).

Kudoh et al conducted a randomized crossover trial evaluating oral rehydration solution for chronic dehydration after proctocolectomy and ileostomy because of ulcerative colitis. Twenty patients, 2–3 weeks postoperatively, alternated 7 days of oral rehydration solution and 7 days of mineral water. Compared with baseline and mineral water, oral rehydration solution increased urinary sodium and reduced plasma renin and aldosterone (Table S1).

Migdanis et al performed a randomized controlled trial examining 40 days of isotonic oral rehydration solution vs standard care after ileostomy for cancer, Crohn disease, ulcerative colitis, or diverticulitis. Thirty‐nine patients received oral rehydration solution after ileostomy, 41 received no oral rehydration solution after ileostomy, and 37 served as non‐ileostomy surgical controls. By postoperative day 20, patients who did not receive oral rehydration solution following ileostomy exhibited higher serum creatinine, lower estimated glomerular filtration rate, and lower serum sodium than the cohort that received oral rehydration solution and the non‐ileostomy control cohort. Although it improved by day 40, elevated creatinine and hyponatremia persisted (Table S2).

Rud et al^28^ conducted a randomized, double‐blinded crossover trial comparing iso‐osmolar and hyperosmolar oral rehydration solutions in patients with a long‐term ileostomy (mean, 11.5 years postsurgery) with indications including cancer, inflammatory bowel disease, diverticulitis, sigmoid volvulus, Hirschsprung disease, and constipation. Eight patients received each solution for 2 days, separated by a ≥14‐day washout. The iso‐osmolar oral rehydration solution significantly increased urine volume and sodium excretion compared with baseline and the hyperosmolar formulation (Table S3).

Rud et al^29^ repeated the design using whey‐based protein oral rehydration solutions (iso‐osmolar whey isolate, caseinate, or hydrolysate; 500 ml daily) in patients with a long‐term ileostomy (mean, 16 years postsurgery). After sequential 4‐week intervention periods with 2‐week washouts, no differences in urine output or sodium were noted between formulations. However, the whey isolate solution decreased plasma renin (–38 IU/L), aldosterone (–4674 pmol/L), and creatinine (–8.3 µmol/L) and increased eGFR (+2.8 ml/min/1.73 m²) compared with the other protein solutions (Table S4).

### Oral rehydration solution administration may reduce stomal output postoperatively

Four studies evaluated stomal output^27^, ^28^, ^29^ (Table 3).

**Table 3:** Stomal output.

Author	Control stomal output, mean (SD), ml/day	ORS stomal output, mean (SD), ml	*P* value
Rud et al^28^	Data not available	Iso‐osmolar: Δ +66 (median difference from baseline) Hyperosmolar: Δ −85 (median difference from baseline)	Iso‐osmolar: 0.31 Hyperosmolar: 0.31
Rud et al^29^	1410 (247)	Whey isolate: 1330 Caseinate: 1494 Whey hydrolysate: 1338	Whey isolate: 0.39 Caseinate: 0.35 Whey hydrolysate: 0.44
Migdanis et al^27^	1135 (316)	1144 (205)	0.92
Westfall et al^30^	Predischarge: 775.7 (378.5) Postdischarge: 993.2 (355.7)	Predischarge: 625 (444.5) Postdischarge: 890.7 (317.5)	Predischarge: 0.005 Postdischarge: 0.025

Abbreviations: Δ, Change from baseline to post‐ORS; ORS, oral rehydration solution.

In the 2019 and 2024 trials by Rud et al, no significant differences were observed in stomal volume between oral rehydration solution formulations or from baseline. Similarly, Migdanis et al found no postoperative difference in stomal output between patients receiving oral rehydration solution and those following standard dietary guidance for 40 days.^28^, ^29^

Westfall et al conducted a retrospective cohort study comparing outcomes in patients with ileostomy for cancer, Crohn disease, ulcerative colitis, or diverticulitis. Preintervention patients followed an enhanced recovery pathway (hydration instructions and standard fluids), whereas postintervention patients received a 36‐day supply of commercial oral rehydration solution with structured nursing education and early postoperative initiation. Compared with standard care, oral rehydration solution recipients demonstrated significantly reduced stomal output 24 h before discharge and lower average postoperative outputs.

### Oral rehydration solution administration reduces hospital readmissions for dehydration and acute kidney injury

Two studies assessed hospital readmission^27^, ^30^ (Table 4).

**Table 4:** Hospital readmission.

Author	Control readmissions, %	ORS readmissions, %	*P* value
Westfall et al^30^	AKI or dehydration: 45.7 Ileus: 20.1 SSI I or II: 6.5 SSI III: 11.8 Any reason: 24.3	AKI or dehydration: 16.5 Ileus: 52.9 SSI I or II: 0 SSI III: 11.3 Any reason: 10.6	AKI or dehydration: 0.0039 Ileus: 0.029 SSI I or II: 0.282 SSI III: 0.96 Any reason: 0.005
Migdanis et al^27^	Fluid or electrolyte abnormalities: 24 Any reason: 29	Fluid or electrolyte abnormalities (IG, NIG): 0, 0 Any reason (IG, NIG): 10, 8	Fluid or electrolyte abnormalities: 0.001 Any reason: 0.02

Abbreviations: AKI, acute kidney injury; IG, intervention group; MW, mineral water; NIG, nonintervention group; ORS, oral rehydration solution; SSI, surgical site infection.

In Westfall et al, oral rehydration solution use lowered readmission rates for dehydration or acute kidney injury relative to the enhanced recovery cohort. Patients receiving oral rehydration solution also had fewer dehydration‐related emergency visits and fewer readmissions for other causes.

Migdanis et al reported higher 30‐day readmission rates for fluid or electrolyte abnormalities in patients with nonoral rehydration solution ileostomy compared with both oral rehydration solution ileostomy and nonileostomy groups.

### Oral rehydration solution use is inconsistent when prescribed but can improve with patient understanding of dehydration risks

Three studies addressed qualitative aspects of oral rehydration solution.^26^, ^29^, ^31^

Kudoh et al collected patient feedback on oral rehydration solution vs mineral water at days 0, 7, and 14, noting comparable tolerability and no reported adverse effects such as dizziness, palpitations, or discomfort.

Rud et al recorded qualitative assessments of protein‐based oral rehydration solutions; whey isolate was rated highest for palatability and aftertaste, followed by caseinate and hydrolysate.^29^

Bradley et al performed semistructured interviews evaluating barriers and facilitators to adherence among participants from the trial of Westfall et al. Five themes influenced adherence: (1) patient‐related factors—belief in oral rehydration solution efficacy; (2) condition‐related factors—comorbidities and hydration habits; (3) therapy‐related factors—taste and texture preferences; (4) health‐system factors—clarity and consistency of provider education; and (5) social and economic factors—support from family and peers vs cost concerns. Patients who received individualized counseling and understood dehydration risks were most consistent with oral rehydration solution use.

## DISCUSSION

Administration of oral rehydration solution may beneficially influence fluid and electrolyte balance in patients with an ileostomy. Findings from this scoping review suggest that, although the available evidence is limited, small, and methodologically heterogeneous, oral rehydration solution may be associated with improvements in fluid balance, natriuresis, and kidney function in both the early postoperative period and among patients with long‐term ileostomy (>10 years). Furthermore, postoperative use of oral rehydration solution was associated with lower rates of hospital readmission. Further research is needed to better understand the barriers to implementing oral rehydration solutions across diverse patient populations. These may include limited clinician awareness of oral rehydration solution efficacy outside high‐output settings, variability in institutional protocols, and patient‐related factors such as palatability, adherence, and misconceptions about sodium intake. Additionally, cost, access to standardized formulations, and lack of consensus guidelines may further hinder consistent clinical adoption. Addressing these barriers through targeted education, implementation research, and guideline development could facilitate broader, evidence‐based integration of oral rehydration solution into postileostomy care.

Although most postoperative intestinal adaptation occurs within the first 2 years following surgery,^12^ the observed improvements in urine volume after iso‐osmolar oral rehydration solution and in renin, aldosterone, creatinine, and estimated glomerular filtration rate after whey protein–based oral rehydration solution in the trials by Rudd and colleagues^28^, ^29^ suggest that oral rehydration solution administration, even a decade after ileostomy, may confer ongoing physiological benefit. Although these studies demonstrated statistically significant improvements in urine and biochemical parameters, the degree to which these changes translate into clinically meaningful reductions in dehydration episodes or renal impairment remains uncertain. Future investigations should therefore aim to correlate improvements in surrogate measures such as urine sodium, volume, and estimated glomerular filtration rate with patient‐centered outcomes, including hydration status, kidney function preservation, and hospital readmissions.

These findings also raise the question of whether stoma output is the most reliable long‐term indicator of hydration status. Both of the trials by Rudd et al reported improvements in biochemical and urinary indices without significant changes in stoma output, suggesting that traditional metrics may underestimate hydration improvements in patients with a long‐term ileostomy. Further research is warranted to determine whether biochemical and urine‐based markers provide a more clinically sensitive assessment of hydration status across postoperative timepoints.

Controlling stoma output nonetheless remains an essential aspect of postoperative management. Migdanis et al found no difference in stomal volume between patients receiving oral rehydration solution and those given standard dietary advice 40 days postoperatively,^27^ and the trials by Rudd et al similarly found no difference between iso‐osmolar and hyperosmolar or protein‐based oral rehydration solution formulations.^28^, ^29^ However, when oral rehydration solution use was integrated with structured postdischarge follow‐up—through in‐person or telemedicine visits led by enterostomal therapy nurses—Westfall et al observed not only reduced 24‐h stoma output before and after discharge but also fewer hospital readmissions because of dehydration and acute kidney injury. These findings, although preliminary, highlight the potential clinical significance of oral rehydration solution within a comprehensive, multidisciplinary postileostomy care framework and suggest possible cost savings through reduced readmission and complication rates.

Abomhya and colleagues studied the economic impact of patients who were readmitted within 30 days of their ileostomy for dehydration‐induced acute kidney injury and found significantly higher healthcare costs compared with those without acute kidney injury readmission.^33^ The median cost of hospitalization for patients readmitted with acute kidney injury was $140,544 (Interquartile Range, $85,072–$242,059) within their subset of patients studied. This substantial financial burden underscores the economic impact of acute kidney injury following ileostomy, highlighting the importance of preventive measures, such as early oral rehydration solution, to reduce unnecessary healthcare expenditures. From a quality improvement perspective, these data demonstrate the need for standardized protocols to enhance early postoperative care, with a focus on adequate fluid and electrolyte management. Implementing evidence‐based hydration strategies could reduce morbidity related to acute kidney injury and its sequelae, such as chronic kidney disease and bone metabolism disorders (eg, osteoporosis and osteomalacia),^21^, ^34^ thereby improving patient outcomes and reducing hospital readmissions. Incorporating oral rehydration solution into postoperative care pathways for patients with ileostomies represents a strategic opportunity to enhance care quality, reduce patient morbidity, and mitigate financial burdens on the healthcare system. Further economic analyses are also warranted, given the low cost of oral rehydration solution compared with the high cost of readmission, which could incentivize its widespread adoption through formal cost‐effectiveness analysis.

Patient adherence is often influenced by the palatability of products and medications, regardless of their clinical efficacy.^35^, ^36^, ^37^ Rudd et al designed their three protein‐based solutions to maximize palatability and administered them at a smaller volume (ie, 500 ml) in their 2024 trial to minimize patient burden.^29^ These factors, in concert with multiple standardized postdischarge clinic and telemedicine visits led by enterostomal nurses, may have contributed to a significantly greater adherence rate to oral rehydration solution compared with the control group (ie, 35.9% vs 88.8%). However, clinicians should be aware that patients may independently modify administration instructions to suit personal preferences—for instance, by diluting the product or reducing the recommended dose. Such adaptations may impact the effectiveness of the intervention and should be closely monitored during the follow‐up period. Bradley et al demonstrated that social support has consistently been shown to enhance adherence, both by reinforcing instruction compliance and by offering emotional encouragement.^31^ In completing the qualitative questionnaires, participants frequently cited the role of family members, friends, and online communities in facilitating consistent use of the product. Future implementations of similar interventions should consider strategies to involve patient support systems proactively, such as providing educational materials to caregivers, directing patients to reputable online forums, or integrating peer support networks where available. These measures can enhance adherence and optimize intervention outcomes, particularly for patients managing complex or ongoing self‐care routines.

Although promising, the current evidence base has notable limitations. Most studies were small, single‐center trials. Westfall et al involved a larger sample (ie, 312 patients), but their retrospective design and lack of a contemporary randomized control group limit causal inference.^30^ The short duration of interventions and follow‐up (ie, hours to weeks postdischarge) leaves long‐term outcomes unexamined but highlights the need to address the physiological implications of colon removal proactively. No study evaluated patient‐centered outcomes beyond hospitalization (eg, quality of life, long‐term kidney function). Cost‐effectiveness was also not formally assessed, although the reduction in readmissions strongly implies downstream savings. Furthermore, most participants were adults undergoing colorectal surgery; data in other groups (eg, children, temporary ileostomy) are lacking. Finally, variations in solution composition (eg, plain glucose‐saline vs protein‐enriched), concentration (eg, iso‐osmolar vs hyperosmolar), dosing (eg, 500 ml/day vs 1000 ml/day), and timing (eg, immediate postoperative window vs years after surgery) complicate synthesis. Because the comparative effectiveness of these approaches remains unclear, future trials should consider enhancing internal and external validity by standardizing aspects of oral rehydration solution interventions. Taken together, the literature currently provides a preliminary evidence base, but crucial gaps still exist. Ultimately, ongoing research is necessary to refine protocols and establish robust, evidence‐based guidelines for nutrition management in patients with ileostomies.

## CONCLUSION

Perioperative nutrition management has emerged as a promising therapeutic approach for patients undergoing ileostomy, but it requires continued optimization. Oral rehydration solution is a low‐cost and highly efficacious intervention that can be harnessed to decrease complications after ileostomy. Exploring the effect of solution intake on ileostomy‐associated outcomes in humans represents a critical avenue of research with profound implications for clinical practice and therapeutic optimization. As part of this effort, there should also be a focus on using validated approaches to oral rehydration solution administration, including composition, dosing, timing, and adjuvant nutrition considerations. A comprehensive, evidence‐based approach to nutrition support tailored to the unique challenges faced by patients undergoing ileostomy within the perioperative window has the promising potential to improve immediate and long‐term health outcomes and enhance the quality of life for this vulnerable and often overlooked patient population.

## AUTHOR CONTRIBUTIONS

**Austin J. Hoeg**: Writing—original draft; conceptualization; investigation; writing—review and editing; data curation; formal analysis. **Jessica Jaques**: Investigation; writing—review and editing. **Remy Johnson**: Visualization; writing—review and editing. **Megan Kocher**: Methodology; data curation; validation; resources; software. **Alexa Weingarden**: Writing—review and editing. **Byron Vaughn**: Writing—review and editing. **Alexander Khoruts**: Writing—review and editing. **Cyrus Jahansouz**: Writing—review and editing. **Ryan T. Hurt**: Writing—review and editing. **Levi M. Teigen**: Writing—original draft; writing—review and editing; supervision; conceptualization; investigation; formal analysis.

## CONFLICT OF INTEREST STATEMENT

Byron Vaughn: personal fees from Illuma Advisors and grants from Takeda, Roche, Celgene, Diasorin, OpenBiome Foundation, and Nestlé. Ryan T. Hurt: Nestlé—consulting, Zealand Pharma—grants. Levi M. Teigen: Nestlé Health Science—paid speaker and independent contractor (research). The remaining authors declare no conflicts of interest.

## Supporting information

Appendix A Full Search Strategies.

Supplemental Table 1 Kudoh 10.

Supplemental Table 2 Migdanis 10.

Supplemental Table 3 Rud 2019 10.

Supplemental Table 4 Rud 2024 10.
