# Supplementary material for: Oral rehydration solution for the management of fluid and electrolyte disturbances in patients with an ileostomy: A scoping review
Source: JPEN J Parenter Enteral Nutr. 2026 Jan 9;50(3):339–51. doi: 10.1002/jpen.70050 (PMC13047308; doi:10.1002/jpen.70050)
Supplement: Supplementary file 1 — Appendix A Full Search Strategies. [file JPEN-50-339-s005.docx]

**Appendix A: Full Search Strategies**

### Medline

1. exp Ileostomy/ or (exp Surgical Stomas/ and exp Ileum/)

2. (ileostom* or ((stoma or stomas or stomata) and ileum)).mp.

3. 1 or 2

4. exp "Diet, Food, and Nutrition"/ or exp Nutritional Sciences/ or exp Malnutrition/ or exp Food Intolerance/

5. (nutrition* or undernutrition* or malnutrition* or malnourish* or diet* or (food adj2 (avoid* or intoleran* or sensitiv*))).mp.

6. exp Dehydration/ or exp Water-Electrolyte Balance/ or exp Water-Electrolyte Imbalance/ or exp Electrolytes/ or Fluid therapy/ or Rehydration solutions/ or exp Calcium/ or exp Calcium, Dietary/ or exp Sodium/ or exp Sodium, Dietary/ or exp Potassium/ or exp Potassium, Dietary/ or exp Magnesium/ or exp Chlorides/ or exp Phosphates/

7. (Dehydrat* or ((Water-Electrolyte or fluid) adj3 (Balance* or imbalance* or abnormalit*)) or rehydrat* or fluid therap*).mp. [mp=title, book title, abstract, original title, name of substance word, subject heading word, floating sub-heading word, keyword heading word, organism supplementary concept word, protocol supplementary concept word, rare disease supplementary concept word, unique identifier, synonyms, population supplementary concept word, anatomy supplementary concept word]

8. (Hypercalcemia* or Milk-Alkali or Milk Alkali or Hyperkalemia* or Hyperpotassemia* or Hypernatremia* or Hypocalcemia* or Hypokalemia* or Hypopotassemia* or Hyponatremia* or Electrolyte* or Sodium* or Potassium* or Magnesium* or Chloride* or Phosphate* or Calcium*).mp.

9. exp Nutrients/ or exp Iron/ or exp Iodine/ or exp Fluorine/ or exp Cobalt/ or exp Manganese/ or exp Selenium/ or exp Copper/ or exp Zinc/ or exp Thiamine/ or exp Vitamin B 12/

10. (Nutrient* or macronutrient* or micronutrient* or (Trace adj2 (element* or mineral*)) or biometal* or Iron or Iodine or Fluorine or Cobalt or Manganese or Selenium or Copper or Zinc or vitamin* or thiamin*).mp.

11. exp Kidney/ or exp Acute Kidney Injury/ or exp Renal Insufficiency, Chronic/

12. ((kidney or renal) adj3 (function* or disease* or injur* or insufficien* or fail* or impair*)).mp.

13. or/4-12

14. 3 and 13

15. exp adult/ or exp adolescent/ or exp child/ or exp infant/

16. exp adult/

17. 15 not 16

18. 14 not 17

19. exp animals/

20. exp humans/

21. 19 not 20

22. 18 not 21

### Embase

1. exp Ileostomy/ or (exp Surgical Stomas/ and exp Ileum/)

2. (ileostom* or ((stoma or stomas or stomata) and ileum)).mp.

3. 1 or 2

4. exp "Diet, Food, and Nutrition"/ or exp Nutritional Sciences/ or exp Malnutrition/ or exp Food Intolerance/

5. (nutrition* or undernutrition* or malnutrition* or malnourish* or diet* or (food adj2 (avoid* or intoleran* or sensitiv*))).mp.

6. exp Dehydration/ or exp Water-Electrolyte Balance/ or exp Water-Electrolyte Imbalance/ or exp Electrolytes/ or Fluid therapy/ or Rehydration solutions/ or exp Calcium/ or exp Calcium, Dietary/ or exp Sodium/ or exp Sodium, Dietary/ or exp Potassium/ or exp Potassium, Dietary/ or exp Magnesium/ or exp Chlorides/ or exp Phosphates/

7. (Dehydrat* or ((Water-Electrolyte or fluid) adj3 (Balance* or imbalance* or abnormalit*)) or rehydrat* or fluid therap*).mp.

8. (Hypercalcemia* or Milk-Alkali or Milk Alkali or Hyperkalemia* or Hyperpotassemia* or Hypernatremia* or Hypocalcemia* or Hypokalemia* or Hypopotassemia* or Hyponatremia* or Electrolyte* or Sodium* or Potassium* or Magnesium* or Chloride* or Phosphate* or Calcium*).mp.

9. exp Nutrients/ or exp Iron/ or exp Iodine/ or exp Fluorine/ or exp Cobalt/ or exp Manganese/ or exp Selenium/ or exp Copper/ or exp Zinc/ or exp Thiamine/ or exp Vitamin B 12/

10. (Nutrient* or macronutrient* or micronutrient* or (Trace adj2 (element* or mineral*)) or biometal* or Iron or Iodine or Fluorine or Cobalt or Manganese or Selenium or Copper or Zinc or vitamin* or thiamin*).mp.

11. exp Kidney/ or exp Acute Kidney Injury/ or exp Renal Insufficiency, Chronic/

12. ((kidney or renal) adj3 (function* or disease* or injur* or insufficien* or fail* or impair*)).mp.

13. or/4-12

14. 3 and 13

15. exp adult/ or exp adolescent/ or exp child/ or exp infant/

16. exp adult/

17. 15 not 16

18. 14 not 17

19. exp animals/

20. exp humans/

21. 19 not 20

22. 18 not 21

23. limit 22 to "remove medline records"

### Web of Science

Indexes=SCI-EXPANDED, SSCI, A&HCI, CPCI-S, CPCI-SSH, BKCI-S, BKCI-SSH, ESCI, CCR-EXPANDED, IC Timespan=All years

| **#** | **Search Statement** |
| --- | --- |
| # 7 | #6 AND #1 |
| # 6 | #5 OR #4 OR #3 OR #2 |
| # 5 | TS=((kidney or renal) NEAR/3 (function* or disease* or injur* or insufficien* or fail* or impair*) ) |
| # 4 | TS=(Nutrient* or macronutrient* or micronutrient* or (Trace NEAR/2 (element* or mineral*) ) or biometal* or Iron or Iodine or Fluorine or Cobalt or Manganese or Selenium or Copper or Zinc or vitamin* or thiamin*) |
| # 3 | TS=(Dehydrat* or ((Water-Electrolyte or fluid) NEAR/3 (Balance* or imbalance* or abnormalit*) ) or Hypercalcemia* or Milk-Alkali or "Milk Alkali" or Hyperkalemia* or Hyperpotassemia* or Hypernatremia* or Hypocalcemia* or Hypokalemia* or Hypopotassemia* or Hyponatremia* or Electrolyte* or Sodium* or Potassium* or Magnesium* or Chloride* or Phosphate* or Calcium*) |
| # 2 | TS=(nutrition* or undernutrition* or malnutrition* or malnourish* or diet* or (food NEAR/2 (avoid* or intoleran* or sensitiv*) )) |
| # 1 | TS=(ileostom* or ((stoma or stomas or stomata) and ileum)) |

###

### Scopus

( ( ( TITLE-ABS-KEY ( ( kidney OR renal ) W/3 ( function* OR disease* OR injur* OR insufficien* OR fail* OR impair* ) ) ) ) OR ( ( TITLE-ABS-KEY ( nutrient* OR macronutrient* OR micronutrient* OR ( trace W/2 ( element* OR mineral* ) ) OR biometal* OR iron OR iodine OR fluorine OR cobalt OR manganese OR selenium OR copper OR zinc OR vitamin* OR thiamin* ) ) ) OR ( ( TITLE-ABS-KEY ( dehydrat* OR ( ( water-electrolyte OR fluid ) W/3 ( balance* OR imbalance* OR abnormalit* ) ) OR hypercalcemia* OR milk-alkali OR "Milk Alkali" OR hyperkalemia* OR hyperpotassemia* OR hypernatremia* OR hypocalcemia* OR hypokalemia* OR hypopotassemia* OR hyponatremia* OR electrolyte* OR sodium* OR potassium* OR magnesium* OR chloride* OR phosphate* OR calcium* OR "fluid therap*" ) ) ) OR ( ( TITLE-ABS-KEY ( nutrition* OR undernutrition* OR malnutrition* OR malnourish* OR diet* OR ( food W/2 ( avoid* OR intoleran* OR sensitiv* ) ) ) ) ) ) AND ( TITLE-ABS-KEY ( ileostom* OR ( ( stoma OR stomas OR stomata ) AND ileum ) ) ) AND NOT INDEX ( medline )

### Cochrane

#1 MeSH descriptor: [Ileostomy] explode all trees

#2 MeSH descriptor: [Surgical Stomas] explode all trees

#3 MeSH descriptor: [Ileum] explode all trees

#4 #2 AND #3

#5 (ileostom* or ((stoma or stomas or stomata) and ileum))

#6 #1 OR #4 OR #5

#7 MeSH descriptor: [Diet, Food, and Nutrition] explode all trees

#8 MeSH descriptor: [Nutritional Sciences] explode all trees

#9 MeSH descriptor: [Food Intolerance] explode all trees

#10 (nutrition* or undernutrition* or malnutrition* or malnourish* or diet* or (food NEAR/2 (avoid* or intoleran* or sensitiv*)))

#11 MeSH descriptor: [Dehydration] explode all trees

#12 MeSH descriptor: [Water-Electrolyte Balance] explode all trees

#13 MeSH descriptor: [Water-Electrolyte Imbalance] explode all trees

#14 MeSH descriptor: [Electrolytes] explode all trees

#15 MeSH descriptor: [Calcium] explode all trees

#16 MeSH descriptor: [Calcium, Dietary] explode all trees

#17 MeSH descriptor: [Sodium] explode all trees

#18 MeSH descriptor: [Sodium, Dietary] explode all trees

#19 MeSH descriptor: [Potassium] explode all trees

#20 MeSH descriptor: [Potassium, Dietary] explode all trees

#21 MeSH descriptor: [Magnesium] explode all trees

#22 MeSH descriptor: [Chlorides] explode all trees

#23 MeSH descriptor: [Phosphates] explode all trees

#24 (Dehydrat* or ((Water-Electrolyte or fluid) NEAR/3 (Balance* or imbalance* or abnormalit*)) or Hypercalcemia* or Milk-Alkali or "Milk Alkali" or Hyperkalemia* or Hyperpotassemia* or Hypernatremia* or Hypocalcemia* or Hypokalemia* or Hypopotassemia* or Hyponatremia* or Electrolyte* or Sodium* or Potassium* or Magnesium* or Chloride* or Phosphate* or Calcium*)

#25 MeSH descriptor: [Nutrients] explode all trees

#26 MeSH descriptor: [Iron] explode all trees

#27 MeSH descriptor: [Iodine] explode all trees

#28 MeSH descriptor: [Fluorine] explode all trees

#29 MeSH descriptor: [Cobalt] explode all trees

#30 MeSH descriptor: [Manganese] explode all trees

#31 MeSH descriptor: [Selenium] explode all trees

#32 MeSH descriptor: [Copper] explode all trees

#33 MeSH descriptor: [Zinc] explode all trees

#34 MeSH descriptor: [Thiamine] explode all trees

#35 MeSH descriptor: [Vitamin B 12] explode all trees

#36 (Nutrient* or macronutrient* or micronutrient* or (Trace NEAR/2 (element* or mineral*)) or biometal* or Iron or Iodine or Fluorine or Cobalt or Manganese or Selenium or Copper or Zinc or vitamin* or thiamin*)

#37 MeSH descriptor: [Kidney] explode all trees

#38 MeSH descriptor: [Acute Kidney Injury] explode all trees

#39 MeSH descriptor: [Renal Insufficiency, Chronic] explode all trees

#40 ((kidney or renal) NEAR/3 (function* or disease* or injur* or insufficien* or fail* or impair*))

#41 #2 OR #3 OR #4 OR #5 OR #6 OR #7 OR #8 OR #9 OR #10 OR #11 OR #12 OR #13 OR #14 OR #15 OR #16 OR #17 OR #18 OR #19 OR #20 OR #21 OR #22 OR #23 OR #24 OR #25 OR #26 OR #27 OR #28 OR #29 OR #30 OR #31 OR #32 OR #33 OR #34 OR #35 OR #36 OR #37 OR #38 OR #39 OR #40

#42 #1 AND #41

### ClinicalTrials.gov

**Condition or Disease:** (Ileostomy)

**Other terms:** (nutrition OR nutrients OR diet OR dehydration OR electrolytes OR minerals OR micronutrients OR macronutrients OR (trace elements) OR kidney)
