# Supplementary material for: Oral rehydration solution for the management of fluid and electrolyte disturbances in patients with an ileostomy: A scoping review
Source: JPEN J Parenter Enteral Nutr. 2026 Jan 9;50(3):339–51. doi: 10.1002/jpen.70050 (PMC13047308; doi:10.1002/jpen.70050)
Supplement: Supplementary file 2 — Supplemental Table 1 Kudoh 10. [file JPEN-50-339-s001.docx]

| **Supplemental Table 1 (Kudoh et al.)** | | | | |
| --- | --- | --- | --- | --- |
| **Biochemical Measure** | **Group A (ORS Days 1-7, MW Days 8-14), mean (SD)** | ***p* (Group A)** | **Group B (MW Days 1-7, ORS Days 8-14), mean (SD)** | ***p* (Group B)** |
| Urine Volume (mL/day) | Day 0: 569 (258)  Day 7: 616 (395)  Day 14: 589 (382) | Δ Day 0-7: data not available  Δ Day 7-14: data not available Δ Day 0-14: data not available | Day 0: 911 (727)  Day 7: 1449 (1109)  Day 14: 1283 (1133) | Δ Day 0-7: data not available  Δ Day 7-14: data not available Δ Day 0-14: data not available |
| Urine Sodium (mEq/g CRE) | Day 0: 15.2 (11.4)  Day 7: 59.4 (38.7)  Day 14: 32.6 (28.8) | Δ Day 0-7: 0.005  Δ Day 7-14: data not available Δ Day 0-14: data not available | Day 0: 17.4 (12.7)  Day 7: 59.4 (38.7)  Day 14: 103 (62.7) | Δ Day 0-7: 0.39  Δ Day 7-14: 0.012  Δ Day 0-14: 0.001 |
| Plasma Renin (ng/mL/h) | Day 0: 15.9 (8.5)  Day 7: 5.9 (3.5)  Day 14: data not available | Δ Day 0-7: 0.005  Δ Day 7-14: data not available Δ Day 0-14: data not available | Day 0: 11.9 (9.6)  Day 7: 6.2 (3.7)  Day 14: 2.5 (1.5) | Δ Day 0-7: 0.079  Δ Day 7-14: 0.006  Δ Day 0-14: 0.004 |
| Plasma Aldosterone (ng/dL)* | Day 0: 59  Day 7: 17  Day 14: 24 | Δ Day 0-7: data not available Δ Day 7-14: data not available Δ Day 0-14: data not available | Day 0: 46  Day 7: 24  Day 14: 9 | Δ Day 0-7: 0.09  Δ Day 7-14: 0.023  Δ Day 0-14: 0.003 |

| **Legend** | **Table Name** |
| --- | --- |
| ORS | Oral Rehydration Solution |
| MW | Mineral Water |
| SD | Standard Deviation |
| *p* | *p* -value |
| Δ | Change |
| * | Signifies numerical estimate from graphical representation |
