# Supplementary material for: Oral rehydration solution for the management of fluid and electrolyte disturbances in patients with an ileostomy: A scoping review
Source: JPEN J Parenter Enteral Nutr. 2026 Jan 9;50(3):339–51. doi: 10.1002/jpen.70050 (PMC13047308; doi:10.1002/jpen.70050)
Supplement: Supplementary file 3 — Supplemental Table 2 Migdanis 10. [file JPEN-50-339-s003.docx]

| **Supplemental Table 2 (Migdanis et al.)** | | | |
| --- | --- | --- | --- |
| **Biochemical Measure** | **Baseline** | **Day 20** | **Day 40** |
| Serum Sodium (mmol/L), mean (SD) | IG: 141 (2)  CG:140 (2)  NIG: 140 (2)  *p* = 0.79 | IG: 139 (2)*  CG: 136 (6)^  NIG: 140 (3)*  *p* = 0.007 | IG: 140 (3)*  CG: 138 (5)^  NIG: 141 (2)*  *p* = 0.01 |
| Serum Creatinine (mg/dL), mean (SD) | IG: 0.9 (0.2)  CG: 0.9 (0.2)  NIG: 0.9 (0.1)  *p* = 0.6 | IG: 0.9 (0.2)*  CG: 1.5 (0.7)^  NIG: 0.9 (0.2)*  *p* = 0.02 | IG: 1 (0.2)*^  CG: 1.1 (0.4)*  NIG: 0.9 (0.2)^  *p* = 0.04 |
| eGFR (mL/min/1.73 m²), mean (SD) | IG: 80 (16)  CG: 83 (23)  NIG: 83 (13)  *p* = 0.85 | IG: 84 (24)*  CG: 59 (22)^  NIG: 86 (22)*  *p* = 0.001 | IG: 81 (21)*^  CG: 69 (21)*  NIG: 86 (25)^  *p* = 0.047 |

| **Legend** | **Table Name** |
| --- | --- |
| IG | Ileostomy Group |
| CG | Control Group |
| NIG | Non-Ileostomy Group |
| eGFR | Estimated Glomerular Filtration Rate |
| *p* | *p* -value |
| * | Significant difference (p < 0.05) between groups within the measured parameter |
| ^ | Significant difference (p < 0.05) between groups within the measured parameter |
