# Supplementary material for: Oral rehydration solution for the management of fluid and electrolyte disturbances in patients with an ileostomy: A scoping review
Source: JPEN J Parenter Enteral Nutr. 2026 Jan 9;50(3):339–51. doi: 10.1002/jpen.70050 (PMC13047308; doi:10.1002/jpen.70050)
Supplement: Supplementary file 4 — Supplemental Table 3 Rud 2019 10. [file JPEN-50-339-s002.docx]

| **Supplemental Table 3 (Rud et al. (2019))** | | | | |
| --- | --- | --- | --- | --- |
| **Biochemical Measure** | **Δ (Baseline vs. Post-Intervention)** | ***p* (Baseline vs. Post-Intervention)** | **Δ (Iso-Osmolar vs. Hyperosmolar)** | ***p* (Iso-Osmolar vs. Hyperosmolar)** |
| Urine Volume (mL/day), median (range) | Iso-Osmolar: +305 (0-840)  Hyperosmolar: -35 (-350-360) | Iso-Osmolar: 0.02  Hyperosmolar: 0.81 | Δ: +470 (0-780) | 0.02 |
| Urine Sodium (mmol/day), median (range) | Iso-Osmolar: +20 (-19-69)  Hyperosmolar: -7 (-54-3) | Iso-Osmolar: 0.08  Hyperosmolar: 0.09 | Δ: +36 (0-66) | 0.02 |

| **Legend** | **Table Name** |
| --- | --- |
| *p* | *p* -value |
| Δ | Change |
