# Supplementary material for: Oral rehydration solution for the management of fluid and electrolyte disturbances in patients with an ileostomy: A scoping review
Source: JPEN J Parenter Enteral Nutr. 2026 Jan 9;50(3):339–51. doi: 10.1002/jpen.70050 (PMC13047308; doi:10.1002/jpen.70050)
Supplement: Supplementary file 5 — Supplemental Table 4 Rud 2024 10. [file JPEN-50-339-s004.docx]

| **Supplemental Table 4 (Rud et al. (2024))** | | |
| --- | --- | --- |
| **Biochemical Measure** | **Δ (Baseline to Post-Intervention)** | ***p* (Baseline vs. Post-Intervention)** |
| Urine Volume (mL/day), mean (95% CI) | Whey Isolate: -194 (-404-17)  Caseinate: +46 (-165-257)  Whey Hydrolysate: +33 (-185-251) | Whey Isolate: 0.07  Caseinate: 0.62  Whey Hydrolysate: 0.76 |
| Urine Sodium (mmol/day), RR (95% CI) | Whey Isolate: 1.25 (0.44-3.5)  Caseinate: 1.20 (0.5-2.9)  Whey Hydrolysate: 1.09 (0.5-0.9) | Whey Isolate: 0.68  Caseinate: 0.68  Whey Hydrolysate: 0.79 |
| Plasma Sodium (mmol/L), mean (95% CI) | Whey Isolate: +1.0 (-0.2-2.0)  Caseinate: +0.8 (-0.3-1.8)  Whey Hydrolysate: +0.3 (-0.7-1.4) | Whey Isolate: 0.054  Caseinate: 0.14  Whey Hydrolysate: 0.51 |
| Plasma Renin (int.enh./L), mean (95% CI) | Whey Isolate: -38 (-65 to -12)  Caseinate: -10 (-37-17)  Whey Hydrolysate: -13 (-41-14) | Whey Isolate: 0.006  Caseinate: 0.47  Whey Hydrolysate: 0.34 |
| Plasma Aldosterone (pmol/L), mean (95% CI) | Whey Isolate: -4674 (-8536 to -812)  Caseinate: -1983 (-5844-1879)  Whey Hydrolysate: +1279 (-2722-5279) | Whey Isolate: 0.019  Caseinate: 0.30  Whey Hydrolysate: 0.52 |
| Plasma Creatinine (µmol/L), mean (95% CI) | Whey Isolate: -8.3 (-15.6 to -0.9)  Caseinate: -0.8 (-8.1-6.6)  Whey Hydrolysate: +0.7 (-6.9-8.3) | Whey Isolate: 0.029  Caseinate: 0.84  Whey Hydrolysate: 0.85 |
| eGFR (mL/min/1.73 m²), mean (95% CI) | Whey Isolate: +2.8 (0.3-5.4)  Caseinate: +0.2 (-2.3-2.8)  Whey Hydrolysate: +1.1 (-1.6-3.8) | Whey Isolate: 0.033  Caseinate: 0.85  Whey Hydrolysate: 0.41 |

| **Legend** | **Table Name** |
| --- | --- |
| *p* | *p* -value |
| Δ | Change |
| CI | Confidence Interval |
| RR | Relative Risk |
